# Supplementary material for: Structural basis of sodium-dependent bile salt uptake into the liver
Source: Nature. 2022 May 11;606(7916):1015–20. doi: 10.1038/s41586-022-04723-z (PMC9242856; doi:10.1038/s41586-022-04723-z)
Supplement: Supplementary file 1 — Reporting Summary [file 41586_2022_4723_MOESM1_ESM.pdf]

nature portfolio

Corresponding author(s): Nicolas Reyes

Last updated by author(s): Jan 2, 2022

Reporting Summary

Nature Portfolio wishes to improve the reproducibility of the work that we publish. This form provides structure for consistency and transparency in reporting. For further information on Nature Portfolio policies, see our Editorial Policies and the Editorial Policy Checklist.

Statistics

For all statistical analyses, confirm that the following items are present in the figure legend, table legend, main text, or Methods section.

n/a	Confirmed	
☐	☒	The exact sample size (*n*) for each experimental group/condition, given as a discrete number and unit of measurement
☐	☒	A statement on whether measurements were taken from distinct samples or whether the same sample was measured repeatedly
☒	☐	The statistical test(s) used AND whether they are one- or two-sided<br>*Only common tests should be described solely by name; describe more complex techniques in the Methods section.*
☒	☐	A description of all covariates tested
☐	☒	A description of any assumptions or corrections, such as tests of normality and adjustment for multiple comparisons
☒	☐	A full description of the statistical parameters including central tendency (e.g. means) or other basic estimates (e.g. regression coefficient) AND variation (e.g. standard deviation) or associated estimates of uncertainty (e.g. confidence intervals)
☒	☐	For null hypothesis testing, the test statistic (e.g. *F*, *t*, *r*) with confidence intervals, effect sizes, degrees of freedom and *P* value noted<br>*Give P values as exact values whenever suitable.*
☒	☐	For Bayesian analysis, information on the choice of priors and Markov chain Monte Carlo settings
☒	☐	For hierarchical and complex designs, identification of the appropriate level for tests and full reporting of outcomes
☒	☐	Estimates of effect sizes (e.g. Cohen's *d*, Pearson's *r*), indicating how they were calculated

Our web collection on statistics for biologists contains articles on many of the points above.

Software and code

Policy information about availability of computer code

Data collection	SerialEM 3.8.16
Data analysis	cryoSPARC v2 and V3, USF Chimera 1.14, ChimeraX 1.2, COOT 0.8.9.2, Prism 8.0.1, Jalview 2.10.4b1, Phenix 1.18.2, CASTp 3.0, PISA 1.52, Consurf 2016, I-TASSER 5.1, MOLEonline 2.5, Muscle 3.8.31, Psipred 4.02

For manuscripts utilizing custom algorithms or software that are central to the research but not yet described in published literature, software must be made available to editors and reviewers. We strongly encourage code deposition in a community repository (e.g. GitHub). See the Nature Portfolio guidelines for submitting code & software for further information.

Data

Policy information about availability of data

All manuscripts must include a data availability statement. This statement should provide the following information, where applicable:

- Accession codes, unique identifiers, or web links for publicly available datasets
- A description of any restrictions on data availability
- For clinical datasets or third party data, please ensure that the statement adheres to our policy

Structural models of NTCPEM-Np87 and NTCPEM-Nb91 complexes have been deposited in Protein Data Bank (PDB) with accession codes 7PQG and 7PQQ, respectively, and the corresponding cryo-EM maps were deposited in the Electron Microscopy Data Bank (EMDB) under accession numbers EMD-13593, and EMD-13596.

Field-specific reporting

Please select the one below that is the best fit for your research. If you are not sure, read the appropriate sections before making your selection.

☒ Life sciences ☐ Behavioural & social sciences ☐ Ecological, evolutionary & environmental sciences

For a reference copy of the document with all sections, see nature.com/documents/nr-reporting-summary-flat.pdf

Life sciences study design

All studies must disclose on these points even when the disclosure is negative.

Sample size	No statistical methods were used to predetermine sample size. Functional experiments were done in at least n=3 biologically independent experiments. Sizes of the cryo-EM data sets were predetermined based on microscope availability, and data collected was sufficient to obtain maps with the reported resolutions that enable structure determination.
Data exclusions	No functional data was excluded from the analyses. Poor-quality cryo-EM micrographs and particles were removed using standard analysis software and protocols
Replication	Functional studies were repeated during biologically experiments as stated in Figure legends and Methods, and results were replicated reasonably well within the experimental error reported. For structural studies, the quality of purified protein and EM specimen was fully reproducible using at least three independent biological replicates
Randomization	Randomization is not relevant to this study
Blinding	Blinding was not performed, because grouping was not applicable to this study

Reporting for specific materials, systems and methods

We require information from authors about some types of materials, experimental systems and methods used in many studies. Here, indicate whether each material, system or method listed is relevant to your study. If you are not sure if a list item applies to your research, read the appropriate section before selecting a response.

Materials & experimental systems

n/a	Involved in the study	
☐	☒	Antibodies
☐	☒	Eukaryotic cell lines
☒	☐	Palaeontology and archaeology
☒	☐	Animals and other organisms
☒	☐	Human research participants
☒	☐	Clinical data
☒	☐	Dual use research of concern

Methods

n/a	Involved in the study	
☒	☐	ChIP-seq
☒	☐	Flow cytometry
☒	☐	MRI-based neuroimaging

Antibodies

Antibodies used	llama (Lama glama) antibody fragments against NTCPCO (nanobodies or VHH) were created and used in this study
Validation	Nanobody 87 and 91 binding to NTCPEM was assayed by size-exclusion chromatography in detergent solutions, as well as by fluorescence-based methods in HEK293 cells expressing the transporters as shown and described in Fig. 1b, and Fig. 4c of this study. Binding of Nb87 to human NTCPWT was also confirmed in HEK293 cells as shown and described in Fig. 4c

Eukaryotic cell lines

Policy information about cell lines

Cell line source(s)	HEK293F (Thermo Fisher)
Authentication	No cell-line authentication was performed
Mycoplasma contamination	No mycoplasma contamination tests were performed
Commonly misidentified lines (See ICLAC register)	No commonly misidentified cell lines were used
